# Female fertility preservation for family planning: a position statement of the Italian Society of Fertility and Sterility and Reproductive Medicine (SIFES-MR)

**DOI:** 10.1007/s10815-024-03197-4

**Published:** 2024-07-20

**Authors:** Claudia Massarotti, Danilo Cimadomo, Valentina Spadoni, Alessandro Conforti, Carlotta Zacà, Andrea Roberto Carosso, Alberto Vaiarelli, Roberta Venturella, Amerigo Vitagliano, Andrea Busnelli, Mauro Cozzolino, Andrea Borini

**Affiliations:** 1 Physiopathology of Human Reproduction, IRCCS Ospedale Policlinico San Martino, Largo R. Benzi, 10, 16132 Genova, Italy; 2 Department of Neurosciences, Rehabilitation, Ophthalmology, Genetics and Maternal and Child Health (DINOGMI Department), University of Genova, Genova, Italy; 3 IVIRMA Global Reseach Alliance, Genera, Clinica Valle Giulia, Rome, Italy; 4 IVIRMA Global Research Alliance, 9.Baby, Bologna, Italy; 5 Department of Neuroscience, Reproductive Science and Odontostomatology, University of Naples Federico II, Naples, Italy; 6 Obstetrics and Gynecology 1U, Physiopathology of Reproduction and IVF Unit, Department of Surgical Sciences, Sant’Anna Hospital Città della Salute e della Scienza di Torino, University of Torino, Turin, Italy; 7 Unit of Obstetrics and Gynecology, University of Catanzaro “Magna Grecia”, Catanzaro, Italy; 8 First Unit of Obstetrics and Gynecology, Department of Interdisciplinary Medicine (DIM), University of Bari, Bari, Italy; 9 Department of Obstetrics and Gynecology, IRCCS Humanitas Research Hospital, Milan, Italy; 10 Department of Biomedical Sciences, Humanitas University, Pieve Emanuele, Milan, Italy; 11 IVIRMA Global Research Alliance, IVI Roma, Rome, Italy; 12 IVIRMA Global Research Alliance, Fundación IVI-IIS la Fe, Valencia, Spain

**Keywords:** Fertility preservation, Family planning, Ovarian reserve, Oocytes cryopreservation

## Abstract

**Purpose:**

This position statement by the Italian Society of Fertility and Sterility and Reproductive Medicine (SIFES-MR) aims to establish an optimal framework for fertility preservation outside the standard before oncological therapies. Key topics include the role of fertility units in comprehensive fertility assessment, factors impacting ovarian potential, available preservation methods, and appropriate criteria for offering such interventions.

**Methods:**

The SIFES-MR writing group comprises Italian reproductive physicians, embryologists, and scientists. The consensus emerged after a six-month period of meetings, including extensive literature review, dialogue among authors and input from society members. Final approval was granted by the SIFES-MR governing council.

**Results:**

Fertility counselling transitions from urgent to long-term care, emphasizing family planning. Age, along with ovarian reserve markers, is the primary predictor of female fertility. Various factors, including gynecological conditions, autoimmune disorders, and prior gonadotoxic therapies, may impact ovarian reserve. Oocyte cryopreservation should be the preferred method. Women 30–34 years old and 35–39 years old, without known pathologies impacting the ovarian reserve, should cryopreserve at least 12–13 and 15–20 oocytes to achieve the same chance of a spontaneous live birth they would have if they tried to conceive at the age of cryopreservation (63% and 52%, respectively in the two age groups).

**Conclusions:**

Optimal fertility counselling necessitates a long-term approach, that nurtures an understanding of fertility, facilitates timely evaluation of factors that may affect fertility, and explores fertility preservation choices at opportune intervals.

## Introduction

In the year 2022, for the first time since the unification of Italy, births fell below the 400,000 thresholds to 393,000. This will cause, according to the Italian National Institute of Statistics, a population decrease from 54.2 million people in 2050 to 47.7 million in 2070 [1]. These figures may be partly attributed to voluntary avoidance of having children, and partly to the aging of the female population of childbearing age. However, the number of women, particularly those over 35 years of age, seeking fertility treatments, is also on the rise. [2].

The “fertility gap” between the number of children a couple actually have and the number they would like to have is heavily influenced by a complex mix of social, economic and cultural factors whose determinants play outside the reproductive medicine centres. However, fertility professionals are asked more frequently than ever to evaluate patients’ reproductive potential, even before an individual starts trying to conceive. Fertility awareness is seen as a necessary first step in reaching the desired “family plan” but, despite a tendency in several countries towards developing programs for fertility awareness specifically addressed to adolescents and young adults [3], most women report that they received information about fertility essentially from social media and non-specialized web pages, friends and relatives [4]. On the contrary, a proper fertility counselling, organized in a long-term care model, would help women to make conscious choices about reproduction and ultimately to reach the desired family size, either through spontaneous conception, assisted reproduction techniques or fertility preservation procedures, whenever appropriate.

Considering these emerging topics that cannot be ignored by reproductive physicians, the aim of this position statement is to define feasible fertility preservation models of care and to identify patients who could best benefit from them.

## Material and methods

This is a position statement on indications for fertility preservation outside the traditional oncofertility setting presented on behalf of the Italian Society of Fertility, Sterility and Reproductive Medicine (SIFES-MR) by a group of its members. The writing group includes Italian reproductive physicians, embryologists and scientists with expertise in fertility evaluation, fertility preservation, assisted reproduction technologies and laboratory quality management.

The positions stated are based on consensus by the authors, who met over a six-month period, as well as society member consultation with revisions and final approval from the SIFES-MR governing council. Consensus was achieved through review of relevant literature and standards related to fertility preservation along with dialogue and discussion by the authors.

The main objective of this position statement is to provide an ideal framework for fertility preservation outside the standard of care for immediate preservation before oncological therapies. In order to fulfil the main aim, this statement will go through the role of the fertility unit in extensive fertility evaluation, including the evaluation of factors known to reduce the ovarian potential, the best available methods for fertility preservation, and when and to whom they should be offered.

## Discussion

### The “biological ovarian age” concept

The expected cumulative chance of a live birth is key to outline the prognosis of any patient seeking fertility preservation. Time to treatment, woman age and ovarian reserve biomarkers are the possible limiting factors. Indeed, women wishing to preserve their future chance of conceiving might have to play against the clock or, in the worst scenario, they might have to face the absence of time left to allow a non-negligible chance of success. An evidence-based definition of the appropriateness of fertility preservation, should pass through the personalised evaluation of the ovarian function of each woman, which includes her hypothetical chance of obtaining a live birth, based on age and ovarian reserve, and, if relevant, the putative effect of other factors (i.e., autoimmune, genetic, oncologic conditions and their treatments) on both ovarian reserve and oocytes competence. All these factors influence the chances of conception and taken together define the “biological ovarian age” concept.

#### Ovarian reserve testing

Age, other than being a predictor of oocytes’ quality, is the main (physiological) reason for ovarian reserve quantitative decline. Notably, different clinical conditions, including endometriosis and ovarian surgery, autoimmune diseases, genetic diseases, and previous gonadotoxic treatments, may negatively impact on the ovarian reserve. Sometimes, the ovarian reserve is reduced without a clear cause (idiopathic diminished ovarian reserve or premature ovarian insufficiency). For these reasons, the estimation of ovarian reserve, through specific tests, is a crucial step in defining the biological ovarian age, which may or may not conform to the chronological age of the patient [5].

The parameters used in the estimation of the ovarian reserve are either biochemical (follicle stimulating hormone, FSH and Anti-Müllerian Hormone, AMH) or morphological (antral follicles count, AFC). FSH plasma concentrations at the start of the menstrual cycle represent a biochemical parameter widely used in the past. This measurement is being gradually abandoned since it shows a wide intra- and inter-cycle variation, and does not show a close correlation with the AFC [6]. Circulating FSH has a significant negative predictive value only with values above 20 mIU/ml [7]. The values of circulating AMH and AFC are instead strongly correlated to the ovarian reserve, reflecting the number of follicles potentially recruitable with controlled ovarian stimulation (COS) [8, 9]. The AFC is measured by transvaginal ultrasonography and it consists in counting the number of small antral follicles (< 10 mm of mean diameter) present in the ovaries in a specific time of menstrual cycles. AFC strongly correlates with AMH circulating concentrations, since it is produced by the same antral and preantral follicles. AFC and AMH are currently the most sensitive indicators for a quantitative evaluation of the ovarian reserve and as predictors of ovarian response to COS. They are not, however, predictive of oocytes’ quality and of the chances of obtaining a spontaneous pregnancy in the short term [9].

### Factors that may influence ovarian age

**Fig. 1:**
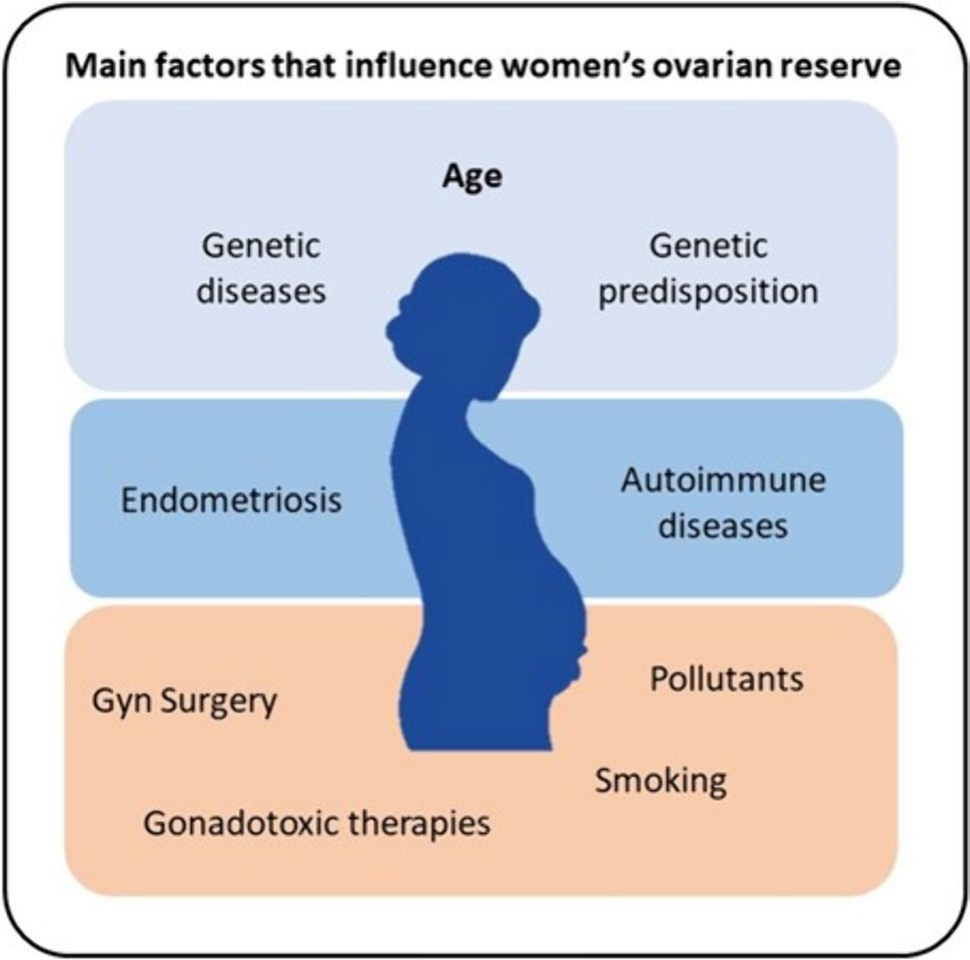
Factors that may influence biological age, reducing the ovarian reserve

Fertility preservation has been traditionally focused on cancer patients at high risk for their reproductive health. However, a wide array of other factors may increase the risk of not being able to reach the desired family size (see Fig. 1).

#### Environment and lifestyle

While a comprehensive discussion of all the lifestyle and environmental factors that may influence fertility is outside the scope of this paper, it's noteworthy to mention some that have demonstrated disruptive effects [10]. Cigarette smoking affects all stages of reproduction, being associated with lower fecundity rates, adverse pregnancy outcomes, and ultimately an earlier onset of menopause [11]. The effects of alcohol on female fertility are more inconsistent and probably a low-moderate use does not affect the ovarian reserve. On the contrary, the effects of alcohol on implantation and pregnancy are well known, and pregnant women should abstain completely from alcohol intake [12]. The use of illicit drugs is related to ovulatory and menstrual disturbances and to adverse pregnancy outcomes [12], but no effect on the age of menopause has been reported. It has to be noted that an abuse of all the mentioned substances might also determine epigenetic changes and DNA damage in germ cells, potentially resulting in inherited imprinting and genetic defects [13].

Endocrine disrupting chemicals (EDCs) may be found in a variety of foods and beverages, in the water and in the air [10]. Bisphenol A (BPA), 2,3,7,8-tetrachlorodibenzo-p-dioxin (TCDD), methoxychlor (MTX), and phthalates have all been demonstrated to interfere with human folliculogenesis, ultimately reducing the follicle pool and potentially causing early ovarian failure [14]. However, the combined toxicity of EDCs on human reproduction, as well as the protective role of the body’s antioxidant systems, is a complex phenomenon, only partially understood that warrants further research.

#### Endometriosis and other benign gynaecological diseases

Various benign gynecological conditions, such as endometriosis or dermoid cysts, can significantly influence ovarian age and consequently affect reproductive potential in multifaceted ways [15]. The ovarian damage may arise from: the effect of time on ovarian reserve as for some diseases, such as uterine myomas, a recovery time is required post their removal before trying to conceive; the direct negative effects of the disease itself (for instance, in endometriosis); and/or the potential iatrogenic consequences resulting from surgical treatment on the ovary, especially when bilateral [15]. However, quantifying the precise reproductive risks posed by each benign pathology or their treatments remains challenging due to the scarcity of reliable data. For instance, any ovarian surgery inevitably compromises a portion of the healthy ovary, leading to an unavoidable reduction in ovarian follicular reserve. This reduction varies depending on factors such as the extent of the pathology, its bilateral nature, and the surgeon's expertise [16].

Among these conditions, ovarian endometriosis and its association with infertility have garnered the most attention in research. Managing endometriosis involves a combination of medications and surgeries aimed at alleviating symptoms and eradicating visible implants. Despite these efforts, the chronic nature of endometriosis and its high recurrence risk often result in repetitive surgeries, potentially culminating in premature ovarian insufficiency (POI) [17]. Furthermore, even in cases where surgery is not pursued, ovarian reserve appears to be decreased in some studies, especially in women with ovarian endometriosis [18]. One possible pathogenic mechanism proposed was follicle depletion due to the excessive activation of primordial follicles triggered by pro-inflammatory pathways such as the PI3K-PTEN-Akt pathway [19]. Moreover, reactive oxygen species (ROS) and proteolytic substances permeating the surrounding tissues were hypothesized to cause the substitution of normal ovarian cortical tissue with fibrous tissue causing follicular loss and intraovarian vascular injury [20].

Available data on fertility preservation in women with endometriosis are scant and support the notion that age would be the most important prognostic factor. However, the cumulative live birth rate (CLBR) seems to be significantly reduced in young women (less than 35 years old) who received ovarian surgery compared to age-matched non-operated women with the disease (CLBR 72.5 vs 42.8% respectively) [21]. These data also show that CLBR is comparable in endometriosis and elective fertility preservation candidates, supporting the available evidence about the quantitative and not qualitative effect of endometriosis on ovarian function [21, 22]. Obviously, women operated two or more times are at higher risk of ovarian failure, and their CLBR decreases together with the reduction of the number of oocytes obtained from a single stimulation [21, 23]. To optimise the chances of reaching the desired family size, fertility should be repeatedly discussed, starting from diagnosis, including the possibilities for fertility preservation, when indicated. According to the available data, the ideal candidates for fertility preservation should be young women with a diagnosis of ovarian endometriosis, before surgery and ideally before age 35, since in this scenario the highest risk of recurrence and the best CLBR coexist.

Fertility counselling is indicated also before ovarian surgery for reasons other than endometriosis, especially when there is a chance of recurrence and/or bilaterality. Dermoid cysts (or mature teratomas) represent up to 70% of benign ovarian tumours in women under the age of 30; the total recurrence rate following cystectomy is 11% [24]. In 90% of the cases, the cysts are unilateral and about 1–2% may undergo malignant transformation [25]. No data are available on the risk for future infertility in this population. However, given their frequency and risk for multiple ovarian cystectomies, an estimation of individual risk of fertility loss should be proposed.

#### Autoimmune diseases

Autoimmune diseases (AD) affect approximately 5% of the population with a clear gender bias, occurring at a rate of 2 to 1 amongst women [26]. Importantly, many of these conditions often first manifest or are diagnosed during reproductive age, with possible significant implications for fertility and pregnancy outcomes [27–29].

The relationship between AD and fertility is highly heterogeneous, varying from one pathology to another and within each individual case. Generally, patients with AD are at a higher risk of infertility and tend to have lower parity compared to the general population. [30, 31]. Several putative mechanisms have been advocated. Firstly, specific autoimmune disorders carry an increased risk of POI, which can be either idiopathic, part of an autoimmune polyglandular syndrome [32] or iatrogenic, as a consequence of gonadotoxic treatments (for example cyclophosphamide for systemic lupus erythematosus and vasculitis [33, 34] or the autologous hematopoietic stem cells transplantation for multiple sclerosis [35]). Furthermore, these patients are more likely to experience recurrent miscarriages, preterm birth, and other obstetrical complications, compared to the general population [36], the most known and studied association being with antiphospholipid syndrome [37].

A second factor contributing to poor reproductive outcomes in patients with AD is the "time window" in which they may be required to postpone pregnancy. This circumstance may arise due to the requirement for invasive investigations, during which pregnancy is contraindicated, or the need to attain disease stabilisation before actively pursuing pregnancy [28]. As a result, there can be a considerable duration in which patients are unable to fulfil their desire for pregnancy, despite their longing to do so.

The presence of AD is also known to lead to sexual dysfunction due to chronic fatigue, pain, anxiety, depression, negative body image and reduced libido [38]. These effects can be related to the disease itself and/or be a consequence of pharmacological treatments used to manage the condition. Lastly, certain AD are associated with other causative conditions of infertility. For example, there is a high correlation between multiple sclerosis and endometriosis, both of which can contribute to fertility challenges in affected individuals [39].

It is important to note that the impact of autoimmune disorders on fertility is complex and multifaceted, and the specific effects can vary widely depending on the individual and the autoimmune condition they have. Hence, it is essential to conduct comprehensive reproductive counselling at the time of an AD diagnosis, providing patients with insightful information regarding the potential reproductive risks they may face in the future. When appropriate, the potential benefits of oocyte cryopreservation should be discussed. This counselling should be personalised, considering the individual's specific characteristics such as age, partner status (if any), pregnancy desire and any existing comorbidities.

#### Genetic disorders

Several genetic disorders result in a diminished ovarian reserve and therefore could represent an indication for fertility preservation [40]. Together with the fertility evaluation, a preconception genetic counselling regarding the chances of transmissibility of the genetic disease/predisposition to the offspring and possibilities and limits of preimplantation genetic testing for monogenic diseases (PGT-M) and prenatal diagnosis (when applicable), is recommended [40, 41].

Turner syndrome (TS) or monosomy X is a chromosomal disorder affecting approximately 1 in 2,500 live-born females [42]. Only 2–5% of the affected women has regular menstrual cyclicity and the chance to obtain spontaneous pregnancies [43]. Indeed, women with full 45 X genotype usually reach POI as adolescents, with small fibrous ovaries. On the other hand, in TS mosaic genotype a residual ovarian activity could be observed through adolescence and early adulthood [44]. The crucial issue is to identify women with residual ovarian function that could be candidates for fertility preservation and to define the perfect timing for it. AMH represents a promising marker of ovarian function in TS women [45]: women with AMH below 8 pmol/l are at increased risk of POI with a sensitivity and specificity of 96% and 86%, respectively [45]. AMH levels correlated also with breast development and spontaneous menarche [46]. In prepubertal girls, ovarian tissue cryopreservation through the removal of an entire ovary may represent an option for future fertility preservation [47]. In post pubertal women oocytes cryopreservation represents another valid option: a few case series confirmed satisfactory results, with a range of mature oocytes of 8.1 ± 3.4 [48]. A careful preconceptional evaluation of TS women should be carried out taking into account that there is an increased risk of endocrinological disorders, hypertensive disorder and diabetes [49]. Cardiac evaluation and the assessment of aortic dissection risk is strongly recommended in women with TS: an aortic size index above 2 cm/m^2^ is a contraindication for pregnancy [50].

X fragile premutation (FMR1) consists in the expansion of CGG repeat to 55 to 199 copies in untranslated FMR1 genes [51] and is associated with a high risk of infertility and POI [52]. In women with FMR1 with an adequate ovarian reserve at the time of the consultation, oocytes cryopreservation could be proposed despite the very few data available in literature [53]. Interestingly, a retrospective analysis of 18 carriers of FMR1 premutation showed a positive correlation between CGG repeats and the number of oocytes retrieved [54]. PGT should always be offered in this condition to avoid full X fragile disorder in the offspring. Pregnancy outcomes in women with FMR1 premutation seems comparable to the general population [55].

Galactosemia is a rare, hereditary disorder of carbohydrate metabolism that affects the body's ability to convert galactose to glucose. It was estimated that more than 70% of women with this condition are at risk of POI at a mean age as young as 13 years [56]. Despite this, natural conception is not impossible: literature shows how it may happen in up to 40% of cases within one year from the POI diagnosis [57, 58]. Fertility preservation through oocytes cryopreservation could be offered to women not desiring a pregnancy at the time of the consultation, despite some studies reporting a reduced response to gonadotropin in women with classic galactosemia compared with age-matched controls [52]. In young prepuberal women ovarian tissue cryopreservation is the only option, with few cases reported in literature, however the patient should be informed that transplanted tissue will face premature functioning failure due to the primary disease [52, 59]. Few data are available about maternal and neonatal outcomes of pregnancies in affected women. In particular the role of galactose metabolites on cognitive long-term functioning of children has not yet been fully investigated [57].

It is well established that BRCA 1–2 mutated carriers are at risk of breast and ovarian cancer. The current guidelines recommend prophylactic bilateral oophorectomy by the age of 40–45 years of age and the fertility consultation should take place before the occurrence of cancer [60]. Cryopreservation of oocytes is an established procedures in these women with good outcome and the possibility to perform PGT-M to prevent transmission in the offspring [60] Ovarian response seems to be similar comparing BRCA carriers to non-carriers [61]. On the other hand, there are conflicting evidence regarding the impact of BRCA mutation on ovarian reserve [62, 63]. Pregnancy appears to be safe in BRCA mutated carriers, even after a previous breast cancer, and does not affect their oncological prognosis [64].

#### Idiopathic premature ovarian insufficiency (POI)

POI affects approximately 1% of the population and, while it can be related to many etiological factors, such those discussed above, in the majority of cases POIs are idiopathic [65].

Some irregular and unpredictable ovarian activity can occur in up to 25% of these women, mainly within one year of diagnosis, with pregnancy reported in up to 5% of cases [66]. However, it is crucial to understand that when there is clinical evidence of POI, the opportunity for fertility preservation has probably already expired since its success depends on the number of oocytes retrieved [67].

Cryopreservation of oocytes, embryos or ovarian tissue can be considered when the risk of POI is assessed early, however, safety and efficacy data lacks in this population [66]. Ovarian tissue cryopreservation may be a successful strategy since it enables fertility preservation at a very young age, including prepubertal girls, and ovarian function restoration for a few years [68]. However, mild clinical symptoms (for example, in very young women vasomotor symptoms are usually absent [69]) and a relative lack of awareness makes such an early evaluation difficult [70].

A detailed family history, especially maternal age at menopause, can be useful to rise suspicion, since it has been demonstrated that first-degree relatives of women with POI have an 18-fold increased risk of POI compared with controls relative risk [71]. These data support the hypothesis of a genetic aetiology of POI, in line with an increasing number of studies demonstrating that multiloci analysis could increase the diagnostic power and the accuracy of POI diagnosis up to 75%, in contrast to the current 25% of positive diagnosis obtained by screening few POI genes [71–75].

Women with some risk factors and relatives of women with non-iatrogenic POI who are concerned about their risk for developing POI should be informed that so far there are no validated tests to identify women that will develop POI, and there are no established prevention measures. Fertility preservation represents a promising option in those not desiring children immediately, although studies on this specific population are lacking, and so are data on their CLBR and chances of success after oocytes or ovarian tissue cryopreservation.

#### Gonadotoxic therapies

While fertility counselling is mandatory before every gonadotoxic therapy, cryopreservation procedures are not always feasible. The most frequent reason is the urgency to start therapies together with a compromised general health status that contraindicates a surgical procedure. The example of leukaemia patients is paradigmatic: it is not possible to wait 2–3 weeks for oocyte cryopreservation; thrombocytopenia and lymphopenia cause significant hemorrhagic and infective risk as well as risk of anaesthesia complications; there is a high chance to malignant cells’ spread to the ovary contraindicating, at the actual state of research, ovarian tissue cryopreservation before therapies in most cases. Moreover, some women in which a procedure is not contraindicated may refuse it for various reasons (fear of medicalization, fear of delaying the start of therapies, …) or may not have had access to fertility preservation services. In these cases, there may be the need of discussing and eventually performing a fertility preservation procedure after cancer therapies.

Short term, patients may ask for a fertility preservation procedure right after the first line of chemotherapy or before a second, more gonadotoxic, treatment. Recent chemotherapy targets growing follicles, contraindicating oocytes/embryo cryopreservation right after it for teratogenicity concerns, but ovarian tissue cryopreservation is feasible in these patients. We expect to find low markers of ovarian reserve, especially low AMH, that is known to fall in the first two weeks after chemotherapy initiation to recover at least six months after its end [76]. Nonetheless, the few data we have about ovarian tissue transplantation show similar function recovery rate and pregnancy rates in women who received low gonadotoxicity chemotherapy before cryopreservation compared to those who did not [77]. Coherently, increased apoptosis but no sign of massive follicular activation was described in exposed ovarian tissue [78]. Exposure to regiments with higher gonadotoxicity, such as high doses of alkylating agents used for some first line regimens, raises efficacy concerns, but more data are needed to draw definitive conclusions [79].

In the long term, most gonadotoxic therapies do not cause immediate ovarian insufficiency, but rather reduce ovarian potential. As a result, the reproductive physician may have to counsel young women, with an ovarian reserve significantly diminished compared to what it is expected at their age, but not yet ready to search for a pregnancy, asking for fertility preservation years after the gonadotoxic therapies. COS in cancer survivors is safe for the woman, even after hormone-sensitive cancers [80], but open questions remain about efficacy, both quantitatively and qualitatively. The severe damage to ovarian reserve translates into a poor quantitative response to COS, probably requiring several stimulation cycles to obtain an adequate number of oocytes. Qualitatively, we know that chemotherapy's main targets are growing follicles, with acute DNA damage induced apoptosis [78], therefore women are counselled to wait approximately one year before trying to conceive. After that safety limit, we have several reports of successful pregnancies in cancer survivors [81]. However, possible long-term effects on oocyte quality are not yet completely excluded, with few animal studies suggesting increased rates of aneuploidies and abnormal maturation in the surviving oocytes, especially after cyclophosphamide exposure [82]. Ovarian tissue cryopreservation is instead not a feasible option years after chemotherapy, as its efficacy is negligible in patients with low follicular density. Extensive fibrosis is usually observed 4–6 months after chemotherapy exposure [78]. Other than ovarian function, the counselling should include a comprehensive evaluation about risks of a future pregnancy, especially due to uterine damage after radiotherapy and to anthracycline-related cardiotoxicity.

### Current strategies for fertility preservation: how, where, and for whom

#### Cryopreservation options

##### Oocytes cryopreservation

Cryopreservation of oocytes through vitrification is the standard and first strategy to be offered to all young women wishing to preserve fertility, as endorsed by all international guidelines [40, 83]. One cycle of COS and an oocytes retrieval procedure require at least 2 weeks, a second COS right after the first one (double stimulation, DuoStim) may aid in maximising the number of oocytes obtained [84]. It is important to note that, in a non-urgent setting, multiple cycles through different months may be carried out to increase the number of cryopreserved oocytes.

The chances of live birth with cryopreserved oocytes are dependent on their numbers and quality, and therefore on the patient's age and ovarian reserve [67]. The mean single vitrified-warmed oocyte to live born child efficiency is 6.4%, but it decreases to 2.5%/single oocyte over 40 years, due to the reduced quality (increase in aneuploidies) [67]. The reported utilization rates are low, around 8–10% [85, 86]. A recent paper reported a cumulative LBR of 41.1% in women that only used the cryopreserved oocytes [87]. Another group, comparing the pregnancy and live birth rates in elective fertility preservation and age-matched cancer survivors found better results in the first cohort (respectively 57.7% vs. 35.7% and 68.8% vs. 41.1%) [88]. No increased rates of anomalies were found in babies born from cryopreserved oocytes [89].

In vitro maturation (IVM) of immature oocytes before cryopreservation is currently used both in infertility patients [90] and in urgent Oncofertility procedures [91], with the advantages of avoiding ovarian stimulation. Cases of cryopreservation of oocytes after IVM are reported also in young patients with genetic conditions such as Turner Syndrome, or with POI [92].

Considering the lower success rates of IVM compared to standard IVF/ICSI after ovarian stimulation [93, 94], it is not yet considered a standard choice for the procedures discussed here, which are mostly carried out in an elective setting, but it may have a role is some selected patients [90].

##### Embryo cryopreservation

Embryo cryopreservation is another standard fertility preservation strategy. It requires COS and an oocytes retrieval procedure, but also a partner or a sperm donor to fertilise the collected oocytes. Its safety and efficacy are mainly demonstrated through data collected in the standard clinical practice of fertility units. However, it has to be noted that approximately 80% of the women seeking elective fertility preservation do not have a partner [86] and the majority of those with a partner chose to not fertilise their oocytes before cryopreservation [95]. Among the reasons that discourage patients are the loss of reproductive autonomy and possible issues with the ownership of stored embryos [96]. We do not have enough data regarding embryo cryopreservation for elective reasons but, if we extrapolate usage rates of embryos cryopreserved from fertility preservation before gonadotoxic therapies, we find percentages as low as 10% [97], raising concerns over the destiny of the abandoned embryos. In some countries, such as Italy, embryo cryopreservation for fertility preservation is prohibited by law.

##### Ovarian tissue cryopreservation

Ovarian tissue cryopreservation can be offered as an alternative fertility preservation method. Since 2019, it is a standard option in the United States and Israel [98], while European guidelines still consider it an innovative method [40]. It requires a laparoscopic procedure to collect ovarian tissue (either a whole ovary or ^1^/_2_ to ^1^/_3_ of the ovary) and another one for re-transplantation at an orthotopic or heterotopic site. The tissue is cryopreserved as small cortical fragments of approximately 1 mm thickness. The slow freezing technique is the most used, since vitrification for ovarian tissue is still experimental. Among its advantages, it does not require a COS; it is feasible in pre-pubertal girls; and the re-transplantation restores ovarian function, for a maximum of five years [99]. If the tissue is transplanted in an orthotopic site, the couple can try to conceive both naturally and through IVF. The in-vitro growth of small follicles from ovarian tissue would be a less invasive option, but a successful protocol in humans is not yet available. Another possible option, experimented with success in Oncofertility cases that may find its role also in elective fertility preservation, is to couple tissue cryopreservation with cryopreservation of oocytes matured though IVM at the time of tissue retrieval [100, 101].

A meta-analysis of 34 studies comparing outcomes of oocytes, embryo and ovarian tissue cryopreservation showed a cumulative live birth of respectively 32%, 41% and, for ovarian tissue, 33% (natural conception) and 21% (IVF) [102]. The chances of success depend on patients’ ovarian reserve at cryopreservation [103]. The ESHRE guidelines suggest an age limit of 36 years, because no live birth was reported in women older than 36 years old who cryopreserved ovarian tissue [104]. Since the procedure is not commonly performed, it is rational to organise a hub-and-spoke model with the laparoscopy performed locally and the cryopreservation in few hub laboratories. The FertiPROTEKT network experience showed that overnight transportation is safe, without damage to the tissue [105].

Most of the data published in literature focus on oncological patients who underwent ovarian tissue cryopreservation in an urgent setting and not elective cryopreservation. However, this fertility preservation technique may have a role for example in prepubertal/very young patients with genetic diseases associated to POI [59] or in patients with autoimmune diseases [106]. The possibility of ovarian function resumption after ovarian tissue transplantation made some hypothesize a role in POI for endocrine function restoration even outside pregnancy desire [99], but its efficacy is capped to a maximum of five years (more commonly 1–2 years), while hormone replacement therapy is a less invasive alternative.

#### Clinical and laboratory KPI for centres offering fertility preservation

The centres offering fertility preservation should be subject to a rigorous quality control, for this reason it is crucial to define shared and reliable key performance indicators (KPIs). Such indicators should be quantifiable, reproducible, consistent, and appropriate for defining the efficacy and safety of care. The standardization of parameters would significantly enhance the processes and enable comparisons of results between centres, taking into account the volume of data generated [107]. Multiple clinical and laboratory KPIs have been proposed for the ART clinics and laboratories [108–110], but the absence of standardization represents a limitation in monitoring the outcomes and the overall performance.

Recently SIFES-MR published a statement, together with SIERR (the Italian Society for Embryology, Reproduction and Research), aimed to propose a set of KPIs covering various aspects essential to an ART clinic, including quality control and ongoing monitoring of clinical and embryological characteristics [107]. Each indicator was assigned a score ranging from 1 to 5. Using these scores, a formula was devised that considers all the parameters and their respective weights. This formula allows the calculation of a central performance score (CPS), which categorizes performance as low, average, good, or excellent.

An ART centre engaged in fertility preservation must demonstrate cryopreservation KPIs that meet or exceed the competence values or benchmarks [107]. For female fertility preservation, the main parameters to consider are those regarding the competence in oocyte cryopreservation, specifically vitrification. The Alpha Consensus, published in 2012, outlines KPIs related to cryopreserved oocytes [109]. On note, only morphologically normal MII oocytes are included, assuming that abnormal oocytes, such as those with smooth endoplasmic reticulum discs, are discarded [109]. The identified KPIs encompass morphological survival, fertilization rate, cleavage rate, embryo development, and implantation rate. Slow freezing is now rarely used, with vitrification being the preferred method for oocyte preservation, highlighting the need for a new consensus that includes more detailed indicators and a larger dataset for comprehensive analysis.

#### A proposed criterion to assess the timeliness of fertility preservation

As media attention on declining natality and infertility grows, so does the public awareness regarding fertility preservation options. Nonetheless, there remains uncertainty about the precise criteria for when and for whom fertility preservation procedures should be offered by fertility units. In general, oocyte cryopreservation for specific conditions impacting woman fertility, as well as to counteract the age-related fertility decline, is the first line fertility approach proposed. However, the cost-effectiveness of fertility preservation for individuals or for society is still unclear. Based on the current level of the evidence, although being highly satisfied that they underwent the procedure [86], only about 10% of the women who vitrified their oocytes return to use them [85, 86, 88, 111, 112]. Yet, we think that the low return rate should not affect the decision-making process regarding this highly sensitive topic. In fact, complete data about return rates might require years or decades to be complete, and no study reported the return rate for second children after having conceived spontaneously a first-born. Secondly, a healthy live birth achieved thanks to the cryopreserved material is still an important result, even in a minority of patients [113]. Thirdly, the surplus vitrified oocytes could be donated either to other women or to research, pending an informed consent and (whenever needed) the additional exams required in the standard work-up, thus creating a virtuous cycle [114].

We propose that the appropriateness and timeliness of fertility preservation should be evaluated based on a key question: can oocyte cryopreservation provide the woman at least with the same chance of live birth as if she would try to conceive spontaneously at the time of the procedure?

**Fig. 2:**
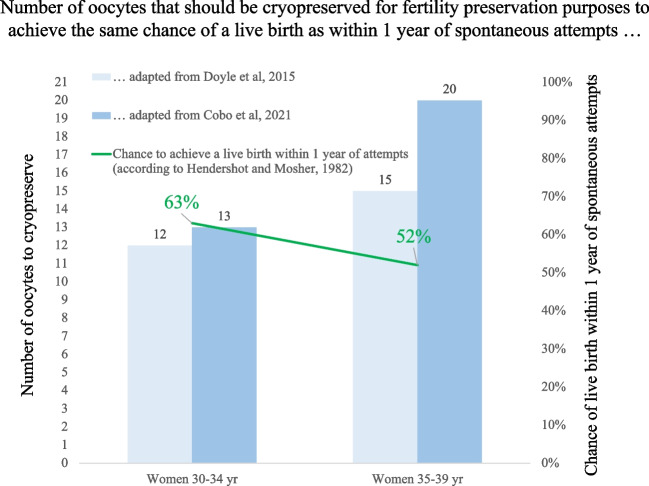
Key question: how many oocytes should we vitrify to provide the woman at least with the same chance of live birth as if she would try to conceive spontaneously at the time of the procedure? The figure reports the number of vitrified oocytes required in women 30–34 years old or 35–39 years old to achieve the same chance of a spontaneous live birth (green line) at the moment of cryopreservation. Data adapted from Hendershot and Mosher, 1982 for spontaneous conception; Doyle et al., 2015 (light blue column) and Cobo et al., 2021 (blue column) for oocyte cryopreservation

Specifically, women fertility follows a U-curve peaking in between 20–30 years to then undergo a decline becoming sharper beyond 35 years [115, 116]. The chance of a healthy couple to conceive within a year of unprotected regular intercourse is estimated to be 63% between 30 and 34 years old, while it decreases to 52% between 35 and 39 years old [117]. To achieve the same 63% chances of a live birth a 30–34 years old woman should vitrify at least 12–13 oocytes; while, in a 35–39 years old woman, the number of oocytes needed to achieve a 52% chance of live birth increases to 15–20 oocytes (see Fig. 2). These numbers are estimated based on the two largest published cohorts: Doyle and colleagues published in 2015 the results of 128 cycles with oocytes previously vitrified for various reasons (elective and medical fertility preservation) [67]; Cobo and colleagues reviewed the results of 641 thaw cycles after cryopreservation for elective reasons [118].

On note, there is insufficient data to make a similar evaluation in women over 40 years old, but we know that as a woman ages, the number of oocytes needed to achieve a live birth increases, while the mean number of oocytes expected to be retrieved during COS steeply decreases, rendering the process less and less efficient [67]. Fertility preservation in women over 40 years old, that are not the ideal target of this procedure, should be evaluated prudently and discussed carefully with the patients due to anticipated poor results in terms of oocyte quality and quantity, as well as the increased obstetric risks associated with maternal age advancement [119]. Furthermore, the few data available in this age group show a reduced likelihood of coming back for utilization [120].

We believe that fertility preservation via oocyte vitrification should be discouraged any time the chance to achieve one live birth is lower than what could be achieved via spontaneous conception. The numbers of oocytes proposed above and in Fig. 2 are to be considered the minimum for a concrete chance to cryopreserve fertility, not a guarantee of future pregnancy. If the patient desires more than one child, the numbers should be at least doubled, as estimated by Doyle and colleagues [67].

These data describe a scenario suitable for fertile or idiopathic infertile patients and without considering the impact of male factor infertility and of the previously discussed factors that may reduce ovarian reserve. In fact, although female age remains the main determinant of oocyte quality, with oocytes’ aneuploidies increasing with age [5], all the factors described above in this manuscript can influence ovarian reserve and move the fertility curve to the right, de-facto advancing patients’ ovarian age. Major events that may influence fertility, such as ovarian surgery for endometriosis or the start of a gonadotoxic therapy for an autoimmune disease, have an impact on fertility that may tip the scale towards fertility preservation in women who might not have strongly considered it otherwise. See Table 1 for a summary of recommendation in patients with the chronic conditions described above. A thorough and patient-centred counselling is therefore imperative to weigh in all factors and thereby either promoting or discouraging the decision to undergo fertility preservation.

**Table 1:** Clinical recommendations by SIFES-MR regarding female fertility preservation in patients with chronic medical condition affecting ovarian reserve

Fertility preservation counselling
Every chronic condition discussed
- Early referral to the reproductive medicine specialist and long-term model of care
Endometriosis and other benign gynecological diseases
- Fertility preservation should always be discussed before surgical procedures on the ovaries - Fertility preservation procedures in women with endometriosis should be carried out preferably before 35 years
Autoimmune diseases
- In case of use of gonadotoxic therapies, fertility preservation procedures should be proposed - However, the possible effects of the ovarian stimulation on the autoimmune disease should be discussed interdisciplinary and with the patient - Obstetrics risks, including risk of miscarriage or preterm delivery, should be discussed during the fertility counselling
Genetic disorders/predisposition
- In case of women affected by genetic disorders at risk of POI at early age (ex. Turner syndrome or Galactosemia), fertility should be discussed starting from prepubertal age - When appropriate, PGT-M and/or prenatal diagnosis to prevent transmission of the genetic disorder/predisposition to the offspring, should be discussed - An evaluation of specific obstetrics risks should be carried out at the time of the fertility counselling (ex. cardiological evaluation in women with Turner syndrome) - We have insufficient evidence regarding a possible reduction of ovarian reserve in BRCA1/2 carriers
Idiopathic POI
- Young women with family history of POI should be referred early to a reproductive medicine specialist for fertility evaluation and counselling regarding fertility preservation - When there is clinical evidence of POI, it may be too late to purse an effective fertility preservation procedure
Previous gonadotoxic therapies
- Ovarian tissue cryopreservation is a feasible opportunity after recent chemotherapy, before more gonadotoxic therapies are administered; while its efficacy is negligible years after therapies when the follicular density is low - At least one year after gonadotoxic therapies, oocytes cryopreservation may be proposed depending on the woman’s residual ovarian reserve, age and ability to sustain a pregnancy - However, we have insufficient data to exclude possible negative effects of previous gonadotoxic therapies on oocyte’s quality and neonatal long-term health

*POI* Premature ovarian failure

Table 2 summarizes the recommendation hereby discussed.

**Table 2:** Clinical recommendations for female fertility preservation by the SIFES-MR

To be effective, fertility counselling should move from an urgent setting to a long-term model of care, in view of a family planning perspective
The current established predictors of fertility preservation’s efficacy are quantitative (age, ovarian reserve measured with AMH and AFC) and qualitative (age)
Conditions such as benign gynaecological diseases, autoimmune and genetic disorders, familiar predisposition, previous gonadotoxic therapies may directly or indirectly affect ovarian reserve reducing it as compared to what is expected based on maternal age
A fertility consultation should always be offered, and a long-term model consent is advisable to promote awareness at different timepoints, such as at diagnosis, before a potentially gonadotoxic intervention (for example ovarian surgery), and throughout the years before fertility decline makes fertility preservation inefficient
Oocyte cryopreservation through vitrification is the gold standard procedure for fertility preservation
Ovarian tissue cryopreservation may have a role in prepubertal and very young girls with genetic diseases linked to premature ovarian insufficiency or in adult women when ovarian stimulation is contraindicated at the time of the procedure (i.e., some autoimmune diseases)
Fertility preservation procedures in an elective setting should be discouraged whenever the chances of live birth would be lower than those the woman would theoretically achieve if trying spontaneously
In women younger than 35 years old, 12–13 oocytes are needed for a 63% chance of live birth, comparable to the expected if the woman would have tried spontaneously at the same age. After 35 years, the number needed for a 52% chance of live birth increases to 15–20 oocytes

#### Strategies to facilitate the decision-making process

Deciding on elective fertility preservation can be challenging for patients. Delaying parenthood by a cryopreservation procedure gives a sense of greater control over reproductive planning, but the medicalization of the reproduction process as well as the uncertainty of the results may be significant stressors. The counselling of women who are contemplating this decision is a critical responsibility for healthcare providers, who must enable an interactive decision-making process and highlight medical complexities, taking into account patients' aims and desires.

The current literature focuses mostly on psychological counselling and the use of supporting materials before urgent fertility preservation in cancer patients [121], but miscommunication may arise also in an not-urgent setting. See Table 3 for some of the possible disconnects between patients and clinicians in this regards, as reported by Drost et al. [122].

**Table 3:** Possible disconnects between providers and patients regarding non-urgent fertility preservation, adapted from Dosrt et al., 2023 [113]

Patients	Clinicians
See elective freezing as a ‘back-up plan’ for delaying	Are hesitant to present elective freezing as a ‘back-up plan’, given the uncertainty of success
Believe ovarian reserve testing to unnecessarily complicate the decision	View ovarian reserve testing as an essential step to make a decision
Express the necessity for a shift in societal attitudes	Express the necessity for a shift in societal attitudes
Desire communality and peer support to assist in the decision-making process	Are sometimes hesitant to recommend community/peer support during the decision-making process

Decision support interventions are needed to improve effective communication to help women navigate toward an informed, values-congruent decision. Among possible interventions there are the creation of patient decision aids (website, apps, leaflets) to make general information more available to women as well as the provision of self-tailored content during consultation with the physician.

## Conclusion

Despite declining birth rates, the demand for fertility evaluations is on the rise, even before a woman contemplates pregnancy. Interest in various fertility preservation techniques is also increasing. In this context, it becomes imperative to define and promote a long-term role for fertility units.

The primary focus of specialists should be on fertility evaluations, with a keen emphasis on promptly identifying factors that may influence a woman's ovarian age, and thereby her risk of subfertility/POI. This risk assessment is dynamic and changes over time, necessitating a long-term care framework. For instance, a patient's need for fertility preservation may vary depending on her age and concurrent conditions, so the advice from fertility specialists may evolve.

The core objective of appropriate counselling is to inform patients, including data about fertility decline with age and the risks linked to pregnancy at advanced maternal age, to raise awareness and ultimately to enable them to make informed decisions about reproduction. In cases where fertility preservation procedures are warranted, the role of fertility counselling is to establish realistic expectations. Patients with very low ovarian reserve should be clearly informed about the expected poor outcomes. Ideally, long-term fertility counselling and care should aim to prevent such occurrences by pinpointing the optimal time for fertility preservation—not too early when it may not be beneficial, and not too late when chances are significantly reduced. However, it's important to acknowledge that not all causes are predictable or preventable.

For these reasons, fertility preservation consultations and, if appropriate, techniques, should be accessible not only for oncologic patients, but for every patient at risk of developing fertility impairment provided that they are given proper counselling on the success rates based on their age. Fertility preservation should gradually move from an urgent-only setting to an elective evaluation that accompanies the woman throughout her life.

## Data Availability

No datasets were generated or analysed in the current manuscript.
